# RNAcode_Web – Convenient identification of evolutionary conserved protein coding regions

**DOI:** 10.1515/jib-2022-0046

**Published:** 2023-08-25

**Authors:** John Anders, Peter F. Stadler

**Affiliations:** Bioinformatics Group, Department of Computer Science, and Interdisciplinary Center for Bioinformatics, Universität Leipzig, Härtelstraße 16–18, D-04107 Leipzig, Germany; Max-Planck-Institute for Mathematics in the Sciences, Inselstraße 22, D-04103 Leipzig, Germany; Institute for Theoretical Chemistry, University of Vienna, Währingerstraße 17, A-1090 Wien, Austria; Facultad de Ciencias, Universidad National de Colombia, Sede Bogotá, Colombia; Santa Fe Institute, 1399 Hyde Park Rd., Santa Fe, NM 87501, USA

**Keywords:** coding sequence detection, comparative genomics, small peptides

## Abstract

The differentiation of regions with coding potential from non-coding regions remains a key task in computational biology. Methods such as RNAcode that exploit patterns of sequence conservation for this task have a substantial advantage in classification accuracy in particular for short coding sequences, compared to methods that rely on a single input sequence. However, they require sequence alignments as input. Frequently, suitable multiple sequence alignments are not readily available and are tedious, and sometimes difficult to construct. We therefore introduce here a new web service that provides access to the well-known coding sequence detector RNAcode with minimal user overhead. It requires as input only a single target nucleotide sequence. The service automates the collection, selection, and preparation of homologous sequences from the NCBI database, as well as the construction of the multiple sequence alignment that are needed as input for RNAcode. The service automatizes the entire pre- and postprocessing and thus makes the investigation of specific genomic regions for previously unannotated coding regions, such as small peptides or additional introns, a simple task that is easily accessible to non-expert users. RNAcode_Web is accessible online at rnacode.bioinf.uni-leipzig.de.

## 1 Introduction

The discrimination of coding and non-coding regions has remained an important task in genome annotation [1, 2]. Three fundamentally different classes of methods have been proposed for this task: comparison with protein databases [3], alignment-free feature-based methods [4, 5] and methods that evaluate multiple sequence alignments (MSAs) [6, 7]. Multiple sequence alignments have also been used to boost the accuracy of gene predictors such as AUGUSTUS [8]. MSA-based methods leverage the characteristic patterns of nucleotide substitutions of evolutionarily conserved coding sequences and thereby gain a sometimes very large advantage in accuracy over pattern-based methods in particular on short coding regions.

The detection of short conserved coding sequences is of practical importance in two quite different application scenarios. Small, independently encoded peptides have received increasing interest following the discovery that some of them carry important physiological functions in both eukaryotes and prokaryotes [9–13]. Despite targeted studies, novel small peptides are still difficult to detect with proteogenomic methods [14]. Many eukaryotic genes, furthermore, exhibit a diverse set of splice variants that are far from completely catalogued. Additional, potentially coding exons thus may have been overlooked if they are not sufficiently long to be detected by gene prediction tools or included in sufficiently abundant RNAs to be discovered in transcriptomics data. In both use cases, alignment-based computational methods are a useful complement to direct experimental data.

The MSAs required as input for comparative methods, however, are time-consuming and often difficult to construct despite recent, major progress [15]. Genome-wide MSAs available through the ENSEMBL and UCSC genome browsers, on the other hand, are restricted to limited sets of species. While the effort of preparing genome-wide MSA containing the species of interest cannot really be spared in genome-scale surveys, the required computational and personnel resources is still prohibitive in case only moderate-size regions, e.g., particular introns or intergenic regions are of interests. Although standard sequence alignment tools can be utilized in such cases, retrieving and aligning the sequences is still a tedious task. The manual work-flow typically comprises the selection of genomes at appropriate phylogenetic distance, a series of blast searches and inspection of their results, retrieval and post processing of the sequences, construction of the MSA, and some quality control on the resulting alignment before the data are suitable as input for a comparative coding sequence detector.

The web service described here is designed to automatize the entire work-flow. Since the advantage of comparative methods is most pronounced for small peptides, special care was taken to ensure that the homology search also works reliably for short query sequences. To this end, an iterative scheme was adopted to identity homologous sequences in a range of sequence similarity at which RNAcode [6] (available from https://viennarna.github.io/RNAcode/) operates with high accuracy. In applications with large queries, such as the scanning eukaryotic introns, the computational costs of the coding sequence detector can become infeasible since the optimization steps in RNAcode’s core algorithm scale quadratically with input size [6]. Large inputs are therefore subdivided into overlapping windows for which MSAs are constructed and scored independently before the results are combined.

## 2 Results

### 2.1 Work-flow

The RNAcode_Web service implements three distinct processing steps: (1) retrieval for suitable sequences, (2) construction of MSAs, and (3) the scoring of MSAs with RNAcode and the collation of the results.

#### 2.1.1 Sequence retrieval and selection

Depending on the size of user’s query sequence, RNAcode_web either runs blastn searches of the query against the NCBI data base, or retrieves plausible candidates from NCBI and generates a temporary local database and runs blastn searches against this local data base. If the NCBI nt database is used, an entrez query is used to identify and discard non-genomic sequences. For long query sequences, a local database is created for efficiency. The query is subdivided into smaller, overlapping chunks (length = 1000 bp) that are searched independently against the local database. This step is parallelized on the server. All blastn hits are then extended to the full length of the query using estimated coordinates. The corresponding sequences are retrieved using blastdbcmd.

The accuracy and sensitivity of RNAcode depends crucially on the pairwise distances of the selected sequences. Therefore, a subset comprising a minimum of 2 and a maximum of 16 extended blastn hits is selected for further processing. Similarity *s* between sequences is computed from a simple pairwise alignment as the number of matches divided by the length of the shorter sequence. The corresponding distance (expressed in percent) is then given as 100(1 − *s*)%. The user may specify the minimal and maximal distance for pairwise alignments; default values are 10 %–60 %. The blastn search is iterated five times with different word lengths (see Table 2 in the Methods section for details) to ensure that distance homologs are included if necessary.

**Algorithm 1**

Density-based clustering algorithm (pseudo code).

**Table:** 

**Input** start_set = {filtered blast results}, min_pair_dist
**Output** reduced_set
1: del_seq = clustered_seq = {}
2: **function** distance(*n*, *m*) ⊳ returns pair-wise distance between two sequences *n*, *m*
3: **function** get_neighbourhood(*s*) ⊳ The neighbourhood always includes *s*
4: neighbours = {}
5: **for** $s^{\prime }\in start\text{\_}set$ **do**
6: **if** $s^{\prime }\notin \text{clustered}\text{\_}\text{seq and}DISTANCE\left(s,s^{\prime }\right)<\min\text{\_}pair\text{\_}dist$ **then**
7: neighbours add *s*′
8: **return** neighbours

9: **while** |clustered_seq|<|start_set| **do**
10: **for** s∈start_set **do**
11: **if** *s* ∉ clustered_seq **then**
12: *N* = GET_NEIGHBOURHOOD(*s*)
13: **if** |*N*| = 1 **then**
14: clustered_seq add *s*
15: **else**
16: centroid = *s*
17: **for** *s*′ ∈ *N* \ *s* **do**
18: *M* = GET_NEIGHBOURHOOD(*s*)
19: **if** |*M*| > |*N*| **then**
20: centroid = *s*′
21: *N* = *M*
22: clustered_candidates add *N*
23: *N* remove centroid
24: del_seq add *N*
25: **return** start_set remove del_seq

The candidates are then subjected to a density-based clustering. Algorithm 1 operates similar to DBSCAN [16] and ensures that representative sequences have a minimum pairwise distance of at least the user-defined threshold (10 % by default) from each other. It determines the centroid of the sequences that best represents the largest number of sequences until the required minimum pairwise distance is reached. The corresponding function reduce_cand_min_dist, which is contained in the module SeqSelection of the Python implementation (see “Software versions and parameters” below), may also be of use in unrelated applications. This step is essential because the data base often contains large numbers of very similar sequences, e.g. from closely related strains or different isolates from the same species. Even large sets of nearly identical sequences, however, convey very little evolutionary information and this cannot be utilized by RNAcode. The final set of sequence is then selected in the order of increasing blastn E-value, i.e., decreasing similarity from the query. The final result is a set of sequences that is as similar as possible to the query given the constraint imposed on their minimal pairwise similarity.

#### 2.1.2 Construction of a MSA and scoring

**Figure 1:**
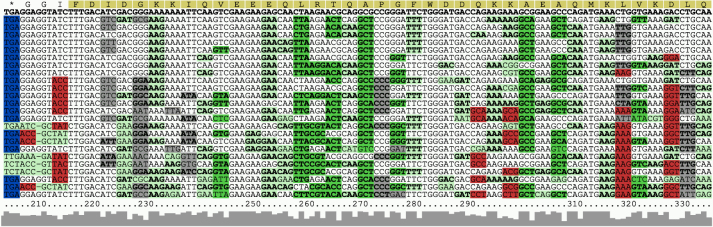
Example of the graphical output produced by RNAcode. The yellow bar on top indicates the predicted coding region, green codons indicate synonymous or similar amino acids, red codons indicate non-conservative substitutions. The level of sequence conservation is indicated as a contour at the bottom. The example shows a novel ORF “nov_131” identified from mass spectrometry data in *Blautia producta* [18]. The alignment contains a diverse set of Bacteriodes species. The image is cropped. The original image with a list of all species can be found in Supplementary Material at http://www.bioinf.uni-leipzig.de/publications/supplements/22-004.

The selected homologs for each query (or sub-query in the case of long input sequences) are the aligned with Clustal Omega [17] and provided to RNAcode for scoring. RNAcode identifies maximal regions with conserved coding potential, which are referred to as “high scoring segments” (HSS) in the following. RNAcode also reports the reading frame (relative to the start position of the input sequence), a p-value for the conserved protein-coding segment, and a graphical representation of corresponding evidence, see Figure 1 for an example.

#### 2.1.3 Output

**Figure 2:**
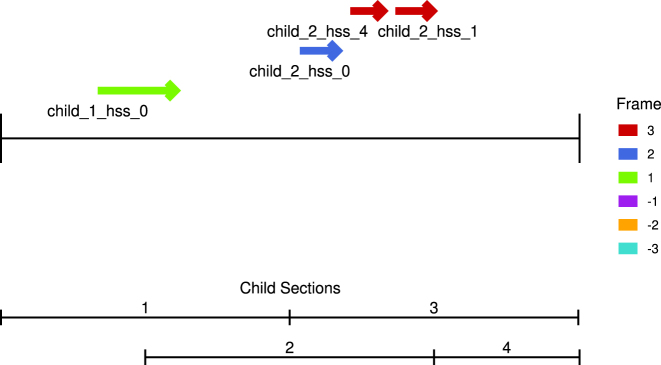
An example for a HSS-plot for the genomic regions harboring the *C. elegans* gene Y54E2A, which encodes a predicted 3′−5′ DNA helicase activity. Each arrow represents one HSS. Colors indicate relative reading frames. The query was subdivided into four sub-queries that were processed separately as indicated at the bottom of the graphic.

The RNAcode_Web service returns a web page with “Coding Regions” and “Alignment Info”. It produces a graphical overview indicating the HSS with a p-value <0.05 that summarizes position, reading direction, and relative reading, see Figure 2. If the query was split into multiple sub-queries, their boundaries are shown at the bottom of the plot.

The output page is interactive and allows the user to apply filters, e.g. a p-value threshold or removing all the best HSS if overlapping HSS in different readings frames are predicted. HSS with high scores tend to show a “shadow” at the opposite strand with a much lower, but still significant score. This is a statistical consequence of the structure of the genetic code. The output echoes the original result table of RNAcode and provides links to annotated alignments, allowing a detailed inspection of the substitutions patters, see Figure 1. An option to export all predicted HSS as protein sequences in fasta format is also available. In case the query was split, overlapping HSS in the same reading frame can be merged into a single prediction.

The output page also provides access to all alignments that were passed to RNAcode for scoring to facilitate the inspection of negative results. The “Alignment Info” pages also show phylogenetic trees constructed by ClustalOmega to identify possible outlying or misaligned sequences.

### 2.2 Example applications

To demonstrate the capability of RNAcode_Web to investigate putative small proteins in prokaryotes, we investigated six candidate peptides (nov_34, nov_102, nov_131, nov_174, nov_180, nov_215) *Blautia producta*, which were found in a metaproteomics survey of an artificial gut community [18]. Figure 1 shows the RNAcode output for nov_131. For all five candidates, RNAcode_Web predicted a conserved coding region with *p* < 0.001 based automatically generated alignments. The corresponding output is available in the Electronic Supplementary Material at http://www.bioinf.uni-leipzig.de/publications/supplements/22-004. Small open reading frames can also be detected efficiently in large, eukaryotic genomes. The human APELA (Apelin Receptor Early Endogenous Ligand), which encodes peptide hormone comprising 54 aminoacids with an intron approximately in the middle of the ORF [19], serves as an example. Here both short coding regions are reliably identified. The full results are available in the Electronic Supplementary Material as well, including a figure of the predicted exons as seen in the UCSC genome browser.

**Figure 3:**
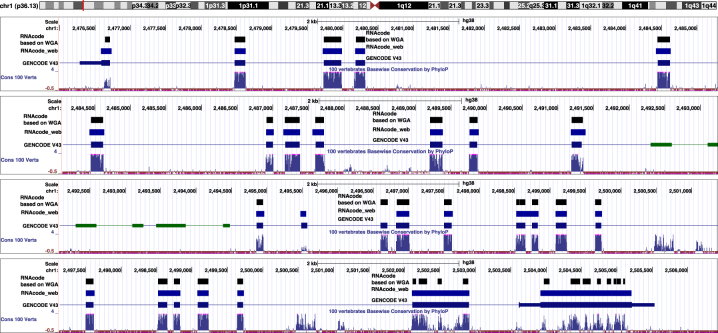
RNAcode_Web predictions for the genomic region harboring the human phosphodiesterase PLCH2. The image was generated by the UCSC browser. For better readability, the region is shown in five slightly overlapping segments. The black “RNAcode WGA” shows the predicted regions by RNAcode based on the whole genome alignment based on 100 vertebrate genomes. The blue track “RNAcode_Web” shows the predicted regions by RNAcode using the alignment constructed by the web service. The histogram track at the bottom shows the conservation level for each base pair across all species in the genome alignment.

RNAcode was originally designed to operate on whole genome alignments (WGA). We compared the results of RNAcode_Web with the results of RNAcode scoring on merged alignment blocks based on the 100 vertebrate WGA provided by the UCSC genome browser. The merging of consecutive alignment blocks is necessary because many blocks in 100 vertebrate WGA are too short for RNAcode to have sufficient statistical power. In order to illustrate the use RNAcode_Web with long inputs we used the DNA sequences of eukaryotic genes as input. The example of the human phosphodiesterase PLCH2 shows that most coding introns are correctly recovered. To compare the results visually, we used blat to map the coding sequences predicted by RNAcode_Web to the human genome. We displayed these data in the UCSC genome browser together with the annotation files generated by RNAcode for the genome wide alignments, see Figure 3. Overall, the RNAcode_Web predictions are more complete, although they have a tendency of extended predicted coding regions a few amino acids beyond the splice sites and sometimes collate consequence exons if they are in frame, i.e., separated by short exons with a length divisible by 3.

Several additional examples are shown in the Electronic Supplementary Material, see http://www.bioinf.uni-leipzig.de/publications/supplements/22-004.

## 3 Discussion

Comparative methods that evaluate substitution patterns by construction require MSAs as input and depend crucially on their quality. This limits their use in practice to application scenarios where suitable alignments are already available, usually in the form of genome-wide alignments, or where can be constructed quickly. The latter is mostly the case if only short, uninterrupted sequences with fairly homogeneous levels of sequences conservation are of interest; for these online blast services are usually sufficient to obtain the required sequences manually. Even in such simple cases, blastn hits often do not extend over the full length of the query and require manual intervention. In general, however, the investigation of a single query that potentially contains multiple coding blocks requires tedious manual data retrieval and multiple steps to prepare the data for scoring, as well as some post processing of the RNAcode results.

In order to alleviate these efforts, we developed a service, RNAcode_Web, that takes a single query sequence as input, which may be short and contain a single coding block or long, encompassing an entire genomic region in a eukaryotic genome. The pipeline then automatically retrieves suitable homologs with pairwise sequence-similarities that both allow reliable alignments and provide enough variation for RNAcode to be a reliable classifier for conserved coding sequence. The pipeline collates the results from RNAcode into a convenient web-based report and provides the pertinent data for download to facilitate further processing. RNAcode_Web is intended to provide its users with fast overview of the conserved coding potential of a DNA sequence of interest, without the need to set up software and download data for comparative analysis locally. Several application examples (accessible in the Electronic Supplementary Material http://www.bioinf.uni-leipzig.de/publications/supplements/22-004) showcase the usefulness of the service.

The RNAcode_Web service is designed to be completely independent of existing sequence annotation and makes only marginal use of external data. It only uses the NCBI taxonomy to restrict the scope of sequence searches in the iterative retrieval step. We argue that the use of taxonomy data is unproblematic since taxonomy information is by definition available for every sequence in the NCBI sequence databases. The taxonomic restriction speeds up the DB search but has no appreciable effect on the final results. The independence from other data sources makes RNAcode_Web applicable without restrictions to sequence data from any organism.

We have developed a rather general approach to automatize the retrieval of sequences with a prescribed minimal similarity to ensure high quality alignments and a prescribed maximal pairwise similarity to ensure that the alignment contains enough sequence variation to be informative. With present implementations of sequence similarity search tools such as blast, it is possible to enforce an upper bound on the similarity only by first retrieving all matches up to a specified minimal similarity. The first step incurs a substantial, but manageable, computational cost whenever the target database contains many highly similar sequences. The MSA is then constructed in subsequent step from the initial set of hits. We believe that this general approach can be transferred also to other applications in comparative genomics. However, the many details, including the bounds on the required sequence similarity, the construction of overlapping or non-overlapping sequence intervals and thus alignment blocks and the size of the aligned blocks will most likely need to be adjusted for specific applications. We have therefore focused here on a specific service rather than a generalist toolkit for sequence retrieval.

## 4 Materials and methods

### 4.1 Data sources

In the current implementation, the service accesses the RefSeq DB “Representative Genome Database” or the nt database “non redundant nucleotide Database” at the user’s discretion, see www.ncbi.nlm.nih.gov/nucleotide respectively www.ncbi.nlm.nih.gov/refseq/. Both data bases are accessed as local copies at the web server and can be updated regularly. Non-genomic sequences—are filtered out during the processing of blastn search results if the nt DB is used. This is done by using an entrez to search the online nuccore DB of NCBI for the GI-identifier from the blastn result and extract the field “Biomol”. Only entries with the value “genomic” in this field are retained. Note that this retrieves only unspliced sequences.

### 4.2 Resource consumption

Typical turn around times for a query range from two to 5 h provided the compute cluster on which the service is hosted is not heavily used by others. This is dominated by the blast searches since a large number of hits (by default 10,000) need to be collected to ensure that not only sequences very similar to the query are retrieved. Current implementations of blast only allow sequence similarity as a lower bound for the search, thus it is not possible to skip near-identical hits. Approximate running times for the examples discussed throughout the text are given together with example outputs in the online supplement. We remark that wall clock times of course may increase if this or other services that share the same dedicated hardware, such as MITOS [20], are under heavy use. In the Electronic Supplementary Material a table with all wall clock times for each of the query examples is given, see http://www.bioinf.uni-leipzig.de/publications/supplements/22-004.

### 4.3 Software versions and parameters

The web server is implemented in python and runs in a flask-environment. A list of all python modules used in the current version is given Table 1. The table also lists the version numbers of tools in use at the time of submission. All code that is necessary to run the web server can be found in the publicly available git-hub repository under https://github.com/JohnBioinf/RNAcode_web.

**Table 1:** Software components for the current implementation of RNAcode_Web webservice.

Software	Information
blast	Version: 2.13.0+
	url: https://ftp.ncbi.nlm.nih.gov/blast/executables/blast+/2.13.0/
ClustalOmega	Version: 1.2.4
	url: http://www.clustal.org/omega/
RNAcode	Version: 0.3
	url: https://github.com/ViennaRNA/RNAcode
mview	Version: 1.67
	url: https://desmid.github.io/mview/
R	Version: 4.1.1
	url: https://cran.r-project.org/src/base/R-4/
ggplot2	Version: 3.3.5
ggrepel	Version: 0.9.1
Cairo	Version: 1.5–12.2
python3	Version: 3.9.10
(webserver)	url: https://www.python.org/downloads/release/python-3910/
filelock	Version: 3.4.2
coolname	Version: 1.1.0
biopython	Version: 1.79
validate_email	Version: 1.3
flask_limiter	Version: 2.1.3
flask	Version: 2.0.2
apscheduler	Version: 3.8.1
ete3	Version: 3.1.2
jinja2	Version: 3.0.3
redis	Version: 4.3.4
python3 (cluster)	Version: 3.7.7
	url: https://www.python.org/downloads/release/377
ete3	Version: 3.1.2
Bio	Version: 1.78

All homology searches use the recent re-implementation of the original blastn algorithm [21] distributed by NCBI. The parameter settings for the blastn searches are discussed in the next section.

The pairwise alignments are computed using the Smith-Waterman algorithm [22] implemented in the python module biopython. By default, we use a simple (Needleman–Wunsch) scoring model that assigns +1 to matches and −1 to mismatches and both gap opening and gap extension. From the alignment, a similarity *s* is extracted as the number of matches divided by the length of the shorter sequences. The correspondence distance between sequences (expressed in percent) is obtained as 100(1 − *s*). Multiple sequence alignments are computed using ClustalOmega. The perl script mview [23] is used to generate a html friendly view of the alignments for the output pages.

The HSS-plot, Figure 2, is produced using R and the library ggplot2.

RNAcode is used with the default parameters. Only the output was set to be tabular and for HSS a plot should be produced.

### 4.4 Parameter setting for blastn searches

Throughout, an E-value cutoff of 0.01 was used. The word size parameters used for blastn during sequence are listed in Table 2. Note that they differ for the construction of the temporary database and the construction of the MSA. While the emphasis in the first case is sensitivity, specificity is the goal for the latter. Since the custom database is very small, also distant homologs can be found reliably already with the first blast search for a subsequence of the query. Thus no iterative search for distant homologs is performed in this case.

**Table 2:** Parameters for blastn searches in different iterations.

Iteration	Word size (build db)	Word size (full pipeline)	Taxonomic restriction
1	14	18	None
2	11	14	Order
3	9	10	Class
4	8	8	Phlyum
5	7	7	Kingdom

In order to speed up the blastn searches, the taxonomic restrictions are enforced in all but the first iteration. To this end, the best hit in the first iteration is used as estimate for the phylogenetic position of the query. The following four iterations then increase both sensitivity and phylogenetic scope.

### 4.5 Construction of a temporary custom database

For long query sequences, RNAcode_Web attempts to retrieve up to 300 sequences from the NCBI sources. For each species, only the best-matching blastn hit is retained. No selection based on similarity to the query is performed at this stage. The target sequences are expanded to five times the query length to compensate for possibly large differences in intron length. The sequences are then converted into a searchable database with blastdbcmd.

## List of abbreviations

NT: nucleotide
ORF: open reading frame
sORF: small open reading frame
DB: data baseid
NCBI: national center for biotechnology information
WGA: whole genome alignment
MSA: multiple sequence alignment
